# A Text Book on Surgery, General, Operative and Mechanical

**Published:** 1887-05

**Authors:** 


A TEXT-BOOK ON SURGERY, GENERAL, OPERATIVE AND MECHANICAL. *By* JOHN A. WYETH, M.D., *Professor of Surgery in the New York Polyclinic, Surgeon to Mount Sinai Hospital, etc., etc. Pp. viii. and 777. New York:* D. APPLETON & CO.

A book can be published, as this one is, without a preface, but such action is so unusual that it is apt to cause unfavorable comment. The fact that this work is sold only by subscription will prejudice some practitioners against it, because its selling price is thus advanced. There are many things in the text which show that the author has not written many books. On the other hand there are many things to be said which are highly complimentary. Among them the following must suffice in this notice. The importance of thorough antiseptic measures is strongly insisted upon, and fairly complete directions are given for their employment. The use of large colored lithographic plates in the chapters on "Ligation of Arteries," "Amputations" and "Hernia," and the use of plates of longitudinal sections of the joints, in the chapter on "Exsections of the Joints," is new. The illustrations throughout the book are good, and the number of them is large. The type is large and the text free from typographical errors. As nearly as one volume can represent it, this one is a very fair exponent of what may be called modern surgery, and will prove an excellent guide to those who use it.

